# Plasmacytoid Dendritic Cells Depletion and Elevation of IFN-γ Dependent Chemokines CXCL9 and CXCL10 in Children With Multisystem Inflammatory Syndrome

**DOI:** 10.3389/fimmu.2021.654587

**Published:** 2021-03-26

**Authors:** Francesca Caldarale, Mauro Giacomelli, Emirena Garrafa, Nicola Tamassia, Alessia Morreale, Piercarlo Poli, Silviana Timpano, Giulia Baresi, Fiammetta Zunica, Marco Cattalini, Daniele Moratto, Marco Chiarini, Elvira Stefania Cannizzo, Giulia Marchetti, Marco Antonio Cassatella, Andrea Taddio, Alberto Tommasini, Raffaele Badolato

**Affiliations:** ^1^ Department of Clinical and Experimental Sciences, University of Brescia, Brescia, Italy; ^2^ ”Angelo Nocivelli” Institute of Molecular Medicine, University of Brescia, ASST Spedali Civili, Brescia, Italy; ^3^ Department of Molecular and translational Medicin and Clinical Chemistry Laboratory ASST Spedali Civili, Brescia, Italy; ^4^ Section of General Pathology, Department of Medicine, University of Verona, Verona, Italy; ^5^ Flow Cytometry & Clinical Chemistry Laboratory, ASST Spedali Civili, Brescia, Italy; ^6^ Clinic of Infectious Diseases, Department of Health Sciences, ASST Santi Paolo e Carlo, University of Milan, Milan, Italy; ^7^ Pediatric Department, IRCCS Burlo Garofolo, Trieste, Italy

**Keywords:** multisystem inflammatory syndrome in children, CXCL9, CXCL10, plasmacytoid DCs, IFN, COVID-19, neutrophil activation

## Abstract

**Background:**

SARS-CoV-2 occurs in the majority of children as COVID-19, without symptoms or with a paucisymptomatic respiratory syndrome, but a small proportion of children develop the systemic Multi Inflammatory Syndrome (MIS-C), characterized by persistent fever and systemic hyperinflammation, with some clinical features resembling Kawasaki Disease (KD).

**Objective:**

With this study we aimed to shed new light on the pathogenesis of these two SARS-CoV-2-related clinical manifestations.

**Methods:**

We investigated lymphocyte and dendritic cells subsets, chemokine/cytokine profiles and evaluated the neutrophil activity mediators, myeloperoxidase (MPO), and reactive oxygen species (ROS), in 10 children with COVID-19 and 9 with MIS-C at the time of hospital admission.

**Results:**

Patients with MIS-C showed higher plasma levels of C reactive protein (CRP), MPO, IL-6, and of the pro-inflammatory chemokines CXCL8 and CCL2 than COVID-19 children. In addition, they displayed higher levels of the chemokines CXCL9 and CXCL10, mainly induced by IFN-γ. By contrast, we detected IFN-α in plasma of children with COVID-19, but not in patients with MIS-C. This observation was consistent with the increase of ISG15 and IFIT1 mRNAs in cells of COVID-19 patients, while ISG15 and IFIT1 mRNA were detected in MIS-C at levels comparable to healthy controls. Moreover, quantification of the number of plasmacytoid dendritic cells (pDCs), which constitute the main source of IFN-α, showed profound depletion of this subset in MIS-C, but not in COVID-19.

**Conclusions:**

Our results show a pattern of immune response which is suggestive of type I interferon activation in COVID-19 children, probably related to a recent interaction with the virus, while in MIS-C the immune response is characterized by elevation of the inflammatory cytokines/chemokines IL-6, CCL2, and CXCL8 and of the chemokines CXCL9 and CXL10, which are markers of an active Th1 type immune response. We believe that these immunological events, together with neutrophil activation, might be crucial in inducing the multisystem and cardiovascular damage observed in MIS-C.

## Introduction

Since December 2019, the epidemic of severe acute respiratory syndrome coronavirus 2 (SARS-CoV-2), causing COVID-19, has rapidly spread worldwide, reaching pandemic proportions in March 2020 (1). Most of the documented cases of COVID-19 have been described in adults; a significant proportion of them present severe pneumonia, often evolving in Acute Respiratory Distress Syndrome (ARDS) (2). Children infected by SARS-CoV-2 rarely develop a severe disease (3), the most common manifestations being fever and cough, often accompanied by influenza-like symptoms, vomiting, and abdominal pain (4). The typical radiographic landmarks of the SARS‐CoV‐2 infection observed in adults may also be present in children, but usually without progressing to the life-threatening complications of COVID-19, described in the oldest patients (5).

COVID-19 adult patients, who develop the most severe disease, usually display a hyper-inflammatory state, resembling secondary hemophagocytic lymphohistiocytosis (sHLH) (6, 7). There is also evidence that critically ill COVID‐19 patients, as measured based on PaO2/FiO2 ratio (<200) (8, 9), display higher plasmatic levels of chemokines (CXCL8, CXCL9, CXCL10), cytokines (IL-6, IL-10), and reactive oxygen species (ROS), bringing attention to the role of the host immune system in developing infection-related complications (10, 11). In adults, sHLH is most commonly triggered by viral infections and occurs in about 4% of sepsis cases (6). Cardinal features of sHLH include unremitting fever, cytopenias, hepatosplenomegaly with liver dysfunction, skin rash, coagulopathy, and hyperferritinaemia, and variable neurologic symptoms; pulmonary involvement (including ARDS) occurs in approximately 50% of patients (6). Thus, ferritin, D-dimer, lactate dehydrogenase (LDH), liver enzymes, and frequent monitoring of the blood cell count have been included in routine laboratory tests, confirming some features of sHLH in critical ill COVID-19 adult patients, although the classic HLH criteria are usually not satisfied (7, 10, 12). Zhou F and colleagues found that the factors associated with admission to the Intensive Care Unit (ICU) and death in a cohort of COVID-19 patients were older age, comorbidity conditions, and elevated body mass index, together with lymphopenia, elevated blood levels of liver enzymes, LDH, D‐dimer, and ferritin (13).

In children, SARS-CoV-2 infection can lead to a hyperinflammatory state with some clinical features of Kawasaki Disease (KD) or even Kawasaki disease shock syndrome. This is probably due to uncontrolled activation of the inflammatory cascade in the course of the immune response against the virus (14). The clinical presentation includes the variable association of persistent fever, mucocutaneous manifestations (polymorphic cutaneous rash, bilateral non-exudative conjunctivitis, peripheral edema, laterocervical lymphadenopathy, and alterations of the lips and/or oral cavity), often with gastrointestinal symptoms. Most of the cases rapidly progress to warm, vasoplegic shock, with hypotension, severe dehydration and cardiac involvement, mainly myocarditis, with heart failure (14, 15). Respiratory manifestations are usually not severe, while many patients show other signs of severe organ involvement, such as acute kidney failure and meningism (16). This syndrome was originally defined as pediatric inflammatory multisystem syndrome temporally associated with SARS-CoV-2 (PIMS-TS) in the United Kingdom (UK) (17, 18) and next as multisystem inflammatory syndrome in children (MIS-C) by the US Center for Disease Control and Prevention (CDC) (19). It was proposed that SARS-CoV-2 infection might function in some children as a trigger for the release of pro-inflammatory cytokines (20).

In this study, we evaluated the levels of pro-inflammatory cytokines such IFN-α, IL-1 and IL-6, chemokines CXCL8, CCL2, CXCL9, and CXCL10, and the immune subsets involved in the anti-viral response in 10 children with COVID-19 as compared to 9 with MIS-C to possibly identify the immunological features underlying such different clinical phenotypes consequent to SARS-CoV-2 infection.

## Methods

### Patients

We retrospectively reviewed 10 patients admitted to the children hospital “Clinica Pediatrica, ASST Spedali Civili” in Brescia from March to April 2020 with a COVID-19 diagnosis. All children suffered from fever, variously accompanied by both respiratory and intestinal symptoms. Nasopharyngeal swab, performed according to CDC guidelines (21), identified the SARS-CoV-2 in all COVID-19 patients. Since middle April, we also identified seven pediatric patients suffering from persistent fever and cardiovascular disease characterized by hypotension, cardiac impairment, all belonging to families with a positive clinical history of SARS-CoV-2 infection. Two additional patients with similar characteristics were diagnosed at the children hospital “Burlo Garofalo” in Trieste. All children fulfilled the diagnostic criteria for MIS-C that were proposed by CDC on May 29^th^ 2020 (19), and retrospectively applied to our patients.

All patients underwent blood sampling and nasopharyngeal swab upon informed consent. Serological testing for COVID-19 was not available in our hospitals at the time of admission.

Full blood count, C reactive protein (CRP), clotting, fibrinogen, ferritin, triglycerides, LDH, troponin, N-terminal pro b-type natriuretic peptide (NT-proBNP) were analyzed at the time of hospital admission, according to our internal COVID-19 diagnostic and therapeutic protocol. Secretions obtained from nasopharyngeal swab were tested for SARS-CoV-2 nucleic acid in children and their caregivers, using reverse-transcriptase quantitative PCR assay (RT-qPCR).

Chest X-ray was performed in all the patients: interstitial pneumonia was diagnosed if bilateral infiltrates and/or areas of ground-glass attenuation were described. All MIS-C patients underwent cardiological evaluation with echocardiography. Cardiac MRI was performed in one patient to better evaluate the myocardial involvement. Clinical features are reported in **Table 1** and **Supplemental Tables 1** and **2**.

**Table 1:** Clinical and laboratory features in COVID-19 and MIS-C at the time of hospital admission.

	COVID-19	MIS-C	
	MEDIAN (IQR)	MEDIAN (IQR)	P-VALUE
Age	2,7 (0.25–11)	10 (4–11)	NS (0.102)
Sex	8 male/2 female	7 male/2 female	NS
Fever	10/10	7/9	NS
Hypotension	0 out 10	7 out 9	**0.0007**
Cardiac involvement	0 out 10	4 out 9	**0.032**
Intestitial pneumonia	7 out 10	7 out 9	NS
Lymphocytes	2018 (1,240–5,457.5)	620 (360–1,370)	**0.014**
Neutrophils	1,995 (1,330–4,440)	4,680 (3,910–8,000)	**0.047**
Neutrophil/ Lymphocyte ratio	1.93	8.92	**0.0039**
Platelets	290,000 (258,000–345,750)	166,000 (113,000–234,000)	**0.0027**
CRP (mg/L)	13,85 (2.55–46.1)	148 (75–227)	**0.0051**
D-Dimer (ng/ml)	483 (377–835)	1107 (685–2,367)	NS (0.2592)
Fibrinogen (mg/dl)	425 (359.5–484)	481 (348–715)	NS (0.2409)
Ferritin (mcg/ml)	290 (136–382)	525 (208–564)	NS (0.1701)
Troponin (ng/L)	8.5 (5–16)	20 (3.5–20.4)	NS (0.2614)
NT-proBNP (ng/L)	113 (64.5–266)	5,970 (615–18,839.5)	NS (0.0946)
IL–6 (pg/ml)	5.2 (4.2–18.3)	133.8 (27.9–171.9)	**0.043**
IFN α (pg/ml)	0.75 (0–3.7)	0 (–)	**0.021**
IFN γ (pg/ml)	0 (–)	0 (0–1.4)	NS (0.084)
TNF (pg/ml)	0 (–)	0 (0–0.3)	NS (0.422)
IL-1β (pg/ml)	0.13 (0–0.9)	0.8 (0.02–1.5)	NS (0.3245)
IL-10 (pg/ml)	2.5 (1.5–3.6)	14.8 (7.1–17.9)	NS (0.1303)
IL-12 (pg/ml)	0 (–)	0.64 (0.3–1.2)	NS (0.1121)
IL-4 (pg/ml)	0 (–)	0 (–)	NS
IL-5 (pg/ml)	0.08 (0–0.3)	0.27 (0.07–0.6)	NS (0.1363)
IL-17 (pg/ml)	0 (0–0.3)	0 (–)	NS (0.2020)
CCL2 (pg/ml)	87.5 (56.7–254.5)	590.4 (161.6–792.9)	**0.008**
CCL5 (pg/ml)	36,135.8 (24,573.5–38,880.9)	12,500.3 (9,091.2–26,586)	**0.015**
CXCL8 (pg/ml)	15.3 (10.7–17.6)	46.9 (21.7–54.4)	**0.018**
CXCL9 (pg/ml)	210.3 (139–304.6)	3,145.5 (1,912.9–5,482.3)	**0.003**
CXCL10 (pg/ml)	812.1 (564.4–4,080.8)	9,339.1 (7,606.5–13,712.8)	**0.018**
MPO (pg/ml)	40 (22–52)	96 (43.5–135.5)	**0.035**
DNA-damage (pg/ml)	21,760 (1,824–22,120)	19960 (17,980–23,780)	NS
ROS (ng/ml)	3.5 (2,93–4,84)	2.7 (1,76–5,01)	NS

The bold values represent statically significant values.

Patient care and research were conducted in compliance with the Case Report guidelines and the Declaration of Helsinki. Local IRB consent was obtained (Ethics Committee of Brescia, protocol NP4000) before recruitment.

### Immunophenotype Analysis

Immunophenotype analysis was performed in all children exept C1, M1, M8, and M9 because of the limited amount of blood obtained at the time of admission. Quantification and enumeration of the major lymphocyte subsets have been obtained labeling 100 μl of fresh blood samples, collected in EDTA as anticoagulant, with the following monoclonal antibodies: anti-CD3-FITC, anti-CD8-PE, anti-CD45-PerCP-Cy5.5, anti-CD19-CD27, anti-CD4-APC, anti-CD16-APC-H7, anti-CD56-Bv421, and anti-HLADR-V500. Samples have been acquired on BD FACSCanto II system and analysis of lymphocyte subsets has been performed using BD FACSDiva™ Software v8. All flow cytometric data have been analyzed with FlowJo software 10.0 (TreeStar Inc.).

### MPO, Damaged-DNA, and ROS Plasma Levels Evaluation

Along with the absolute number of granulocytes, Myeloperoxidase (MPO), ROS, and damaged-DNA have been evaluated in plasma, using enzyme-linked immunosorbent assay (ELISA) techniques. Human MPO Instant ELISA kit, DNA damage competitive ELISA kit (Thermo Fisher Scientific), soluble ROS quantification (LSBio) were measured according to the manufacturer’s instructions.

### Analysis of Type I IFNs-Inducible Genes *via* RT-qPCR

Granulocytes and peripheral blood mononuclear cells (PBMCs), from both patients and healthy donors, have been isolated by Ficoll-Paque PLUS (GE Healthcare) density gradient centrifugation of peripheral blood collected in tube containing heparin as anticoagulant. Four donors for each group have been tested because limited amount of samples has been available for this further analysis.

Erythrocytes present in the granulocyte fraction have been removed by incubation of the cells in a proper lysis buffer at 4°C. The purity of granulocytes has been assessed by cytometric analysis with CD14/CD16/HLA-DR/CD19 markers, obtaining a purity greater than 98% (16+/HLA-DR-/high SSC). Monocytes (CD14+ plus CD14dim/CD16+), instead, were less than 1%.

After washing with cold PBS, both granulocytes and PBMCs underwent to lysis with RLT lysis buffers (Qiagen, Hilden, Germany) and immediately frozen at −80°C until the time of use. Total RNA has been extracted by the RNeasy Mini Kit (Qiagen). To completely remove any possible contaminating DNA, an on-column DNase digestion with the RNase-free DNase set (Qiagen) has been performed during total RNA isolation. Purified RNA has been reverse-transcribed into cDNA using PrimeScrip RT reagent Kit (Takara Bio) and random hexamer primers (Takara Bio), while qPCR has been carried out using TB Green Premix Ex Taq (Takara Bio). Sequences of gene-specific primer pairs are the following: GAPDH, forward AACAGCCTCAAGATCATCAGC and reverse GGATGATGTTCTGGAGAGCC; IFIT1 forward TCATCAGGTCAAGGATAGTCTG and reverse GGTGTTTCACATAGGCTAGTAG; ISG15 forward ACTCATCTTTGCCAGTACAGGAG and reverse CAGCATCTTCACCGTCAGGTC; VCAN forward ATGTCACTCTAATCCCTGTCGT and reverse ATGTCTCGGTATCTTGCTCAC. Data have been calculated by Q-Gene software (http://www.gene-quantification.de/download.html) (22) and expressed as mean normalized expression (MNE) units after GAPDH normalization. Granulocytes purity has been assessed by flow cytometry and resulted typically >98%. In addition, to exclude contamination of granulocyte preparations by monocytes, which have higher level of total RNA and can generate false-positive results (23), we routinely test the mRNA expression of VCAN, a gene highly expressed by monocytes but not granulocytes. Granulocytes samples showing high level of VCAN expression were excluded from further analysis.

### Cytokines and Chemokines Plasma Dosage

The assays have been performed on plasma isolated from whole blood of all the children with COVID-19 and seven out of nine with MIS-C because of the limited amount of plasma available. To eliminate platelets as much as possible, blood in EDTA as anticoagulant was processed immediately after sampling and then centrifuged for 20 min at 2,000 × g at 5°C. The plasma resulting after centrifugation was aliquotated and frozen at −80°C until use.

Plasma levels of CXCL10 (IP-10), CXCL8 (IL-8), CXCL9 (MIG), CCL5 (RANTES), and CCL2 (MCP-1) have been analyzed with flow cytometric bead array method, using the Human Chemokine Kit (Becton Dickinson, San Jose, CA, USA), following the manufacturer’s instructions. IL-1ß, IL-2, IL4, IL-5, IL-6, IL-10, TNF-α, IL-17A, IFN-α, and IFN-γ have been dosed by Flex set custom cytometric bead array technique (Becton Dickinson). Afterwards, data have been acquired on BD FACSCanto II flow-cytometer and analyzed by FCAP v3 software.

### Statistical Analysis

Statistical analysis has been performed *via* GraphPad Prism 5.0 software. Mann-whitney U test has been used to compare COVID-19 samples with MIS-C ones in cytokines and chemokines plasma levels, in mediators of granulocytes activity and in the evaluation of the main laboratory parameters. As regards the Boolean parameters, the Fisher’s exact test has been applied. Finally, Kruskal-Wallis test has been used for real time RT-PCR data analysis. A p-value less than 0.05 is considered significant.

## Results

### Clinical and Laboratory Features

COVID-19 children (mean age 2.7 years old) suffered from respiratory symptoms, such as cough, nasal congestion, and runny nose, but also respiratory distress and difficulty in breathing, as reported by other Authors (2, 4, 24). None of the 10 patients required ICU admission. Only one patient developed moderate respiratory distress, successfully treated with oxygen supplementation through High Flow Nasal Cannulae. Chest X-ray has been performed in all patients, along with the support of lung ultrasound, and thorax CT scan in the most severe patients. Seven out of 10 patients fulfilled the diagnostic criteria for interstitial pneumonia (**Table 1**), similarly to the adult form (2), but milder.

From April until May 2020, we identified nine pediatric patients suffering from hypotension (defined as systolic pressure below 5^th^ percentile per height and age), cardiac impairment, and persistent fever, diagnosed with atypical or complete KD, all belonging to families exposed to SARS-CoV-2. Thus, all patients fulfilled the diagnostic criteria for MIS-C (15). The nasopharyngeal swab was negative in all patients, except in patient M1 and patient M5 (**Supplemental Table 2**).

The median age of patients is higher in MIS-C (10 years old) than in COVID-19 (2.7 years old) (**Table 1**). Besides hypotension and cardiogenic shock, persistent fever and gastrointestinal symptoms were the most common manifestations in patients with MIS-C, similarly to the features reported in other studies (18). Two patients required ICU admission and received inotropes, ACE-inhibitors, and diuretics.

Because at the time of hospitalization the COVID-19-associated multisystem inflammatory disease had not yet been described, these patients were diagnosed with atypical or complete KD, and then received intravenous immunoglobulin (IVIG), steroids, and acetylsalicylic acid (ASA) to prevent further cardiac complications. Kobayashi score was also calculated (see **Supplemental Table 2**), predicting high risk of IVIG-non responders.

Most of the COVID-19 patients showed clinical and radiological features associated with interstitial pneumonia (7 out of 10). By contrast, patients with MIS-C showed cardiac impairment (p = 0.032) and hypotension (p = 0.0007; **Table 1**), shock and reduction of left ventricle ejection fraction. NT-proBNP blood levels were higher in MIS-C but this difference was not statistically significant (median 5,970 ng/L *vs* 113 ng/L in COVID-19 patients, p = 0.094; **Table 1**), while troponin levels were comparable between the two groups.

**Figure 1:**
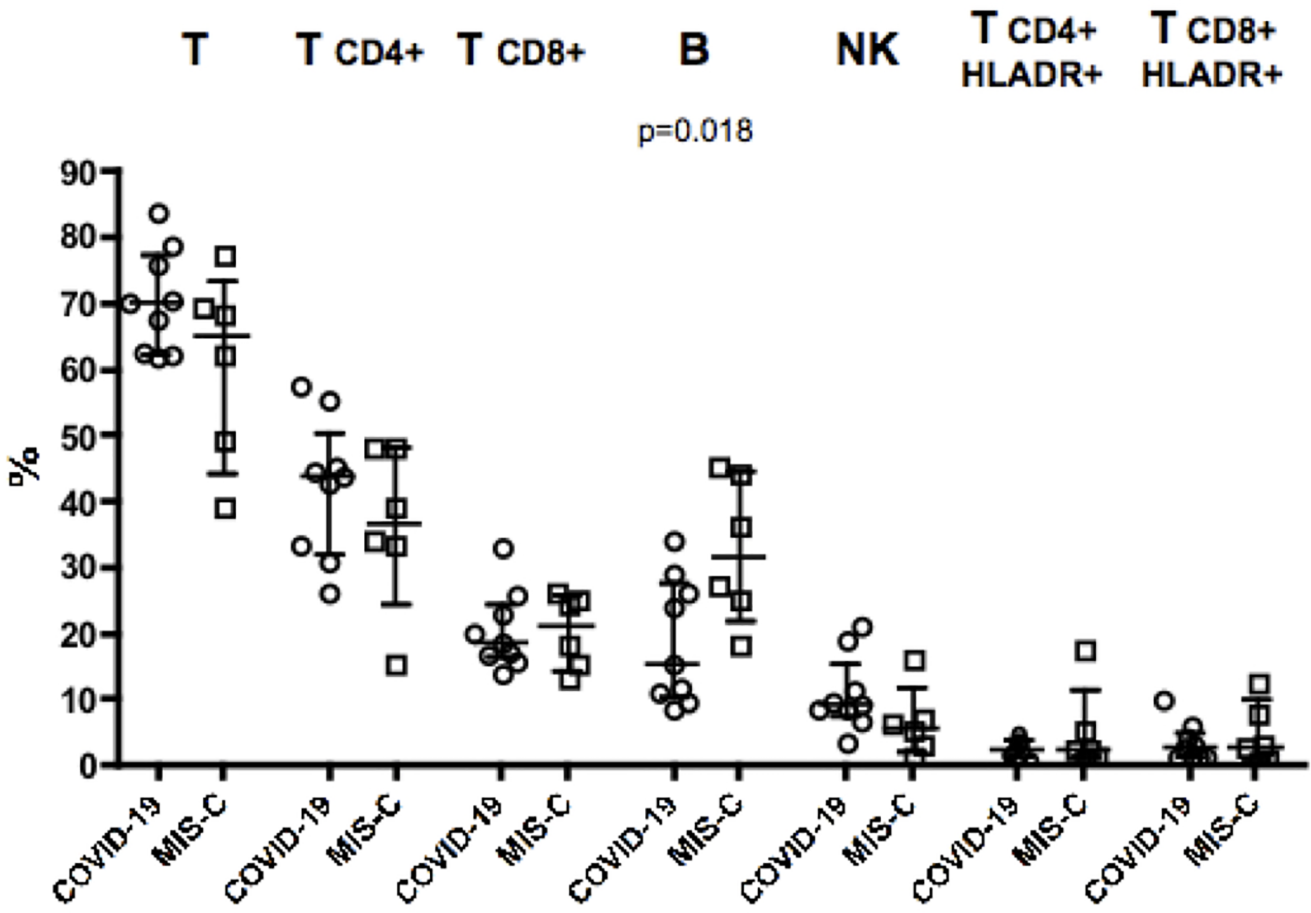
Percentages of major lymphocyte subsets measured in blood of nine children with COVID-19 (dots, C2–C10) as compared to six patients with MIS-C (squares, M2–M7). In the scatter plot graphs, data are presented as median ( ± IQR) for CD3+, CD4+ and CD8+ T lymphocytes, CD19+ B lymphocytes, CD3−CD16+CD56+ NK cells, CD4+HLADR+, and CD8+HLADR+ activated T lymphocytes. Statistical analysis was performed by using the Mann-Whitney U test.

Analysis of blood counts at the time of hospital admission revealed lymphocytopenia (620 cells/µl in MIS-C as compared to 2,018 cells/µl in COVID-19 patients, p = 0.014) and lower platelet counts (166,000/µl in MIS-C than 290,000/µl in COVID-19 children, p= 0.027; **Table 1**) in patients with MIS-C. Analysis of lymphocyte subpopulations at disease onset showed an increased percentage of B lymphocytes in MIS-C (31.5% in MIS-C *vs* COVID-19 patients 15.2%, p = 0.018;, while no significant difference was observed in other lymphocyte subpopulations (**Figure 1**). Neutrophil counts were higher in MIS-C than COVID-19 children (4,680 cells/µl in MIS-C *vs* 2,070 cells/µl in COVID-19, p = 0.047; **Table 1**). In addition, a higher neutrophil/lymphocyte ratio was observed in the MIS-C group as compared to the COVID-19 one (8.92 in MIS-C *vs* 1.93 in COVID-19, p = 0.0039; **Table 1**). We also measured plasmatic levels of MPO, damaged-DNA, and ROS, as markers of neutrophil levels and activity. Patients with MIS-C displayed higher MPO levels than COVID-19 (96 ng/ml *vs* 40 ng/ml, respectively, p = 0.035; **Table 1**). In contrast, analysis of the extent of damaged-DNA, which is a typical component of neutrophil extracellular traps (NETs), and ROS has not revealed significant differences between the two groups (**Table 1**).

Analysis of biochemical markers of inflammation such as CRP, ferritin, and D-dimer showed that CRP plasma levels were significantly higher in MIS-C (148 mg/L) than in COVID-19 children (13.85 mg/L) (p = 0.0051; **Table 1**), while ferritin and D-dimer levels were usually higher in MIS-C than in COVID-19, although the difference was not statistically significant (**Table 1**).

### IFN-α Levels and Expression of Type I IFNs Inducible Genes ISG15 and IFIT1 Were Increased in COVID-19

**Figure 2:**
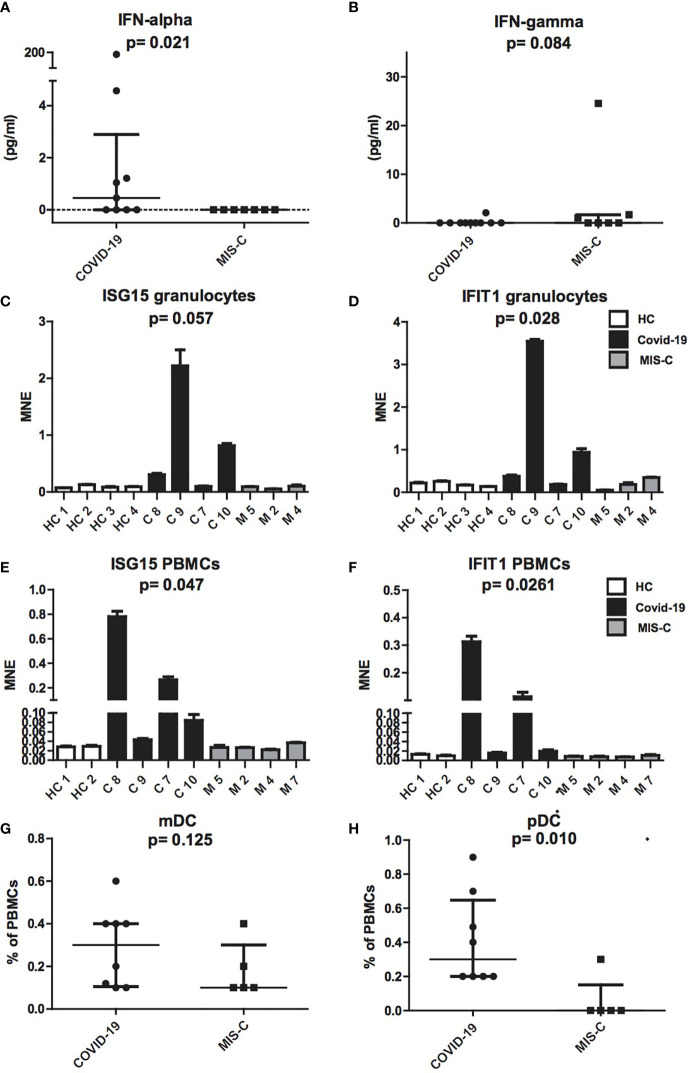
Production of IFN-α and IFN-γ, ISG15 IFIT1 mRNA expression levels and mDCs and pDCs in patients with COVID-19 and MIS-C. **(A, B)**: Plasmatic levels of IFN-α **(A)** and IFN-γ **(B)** in patients with COVID-19 (dots, C1–C10) and MIS-C (squares, M1–M7). In the scatter plot graphs, data are presented as median ( ± IQR). Statistical analysis was performed by using the Mann-Whitney U test. **(C–F)**: ISG15 and IFIT1 mRNA expression levels, as measured by using quantitative RT-qPCR on granulocytes **(C, D)** or PBMCs **(E, F)** of children with COVID-19 (black colums, C1–C4) as compared to MIS-C (gray colums, M1–M4) and adult healthy controls (white columns, HC1–HC4). In the graphs data are presented as median ( ± IQR). Error bars represent standard errors calculated from triplicate qPCR reactions. Statistical analysis to compare ISG15 and IFIT1 expression in the three groups of donors was performed by using the Kruskal-Wallis test. **(G, H)**: mDCs **(G)** and pDCs **(H)** was measured in blood of children with COVID-19 (dots, C1–C8) as compared to MIS-C (squares, M1–M5) and expressed as percentage of PBMCs. In the scatter plots the internal horizontal lines indicate the mean value and the two horizontal lines across the mean indicate the standard deviation. Statistical analysis was performed by using the Mann-Whitney U test.

To investigate the pathogenesis of MIS-C, we comparatively evaluated IFN-α plasmatic levels and the extent of type I IFNs inducible genes mRNAs from granulocytes and PBMCs in children with MIS-C and COVID-19 (**Figure 2**). Comparison between the two groups revealed a significant increase of IFN-α levels in COVID-19 children (0.75 pg/ml) as compared to MIS-C (p = 0.021; **Figure 2A**). In contrast, IFN-γ was undetectable in the plasma of both groups (**Figure 2B**).

Analysis of type I IFNs inducible genes expression performed in granulocytes and PBMCs showed increased transcription of ISG15 and IFIT1 mRNAs in cells of the COVID-19 patients, while ISG15 and IFIT1 mRNAs were detected in MIS-C at levels comparable to adult healthy controls (**Figures 2C–F**).

Because plasmacytoid dendritic cells (pDCs) constitute the main source of IFN-α in blood (25, 26), we assessed the numbers of pDCs and myeloid DCs (mDCs). Interestingly, analysis of mDC and pDC counts revealed a profound depletion of pDCs in patients with MIS-C (p = 0.01; **Figure 2G**), as compared to children with COVID-19, while mDCs reduction was not statistically different (**Figure 2H**).

### Differences in Inflammatory Chemokines and Cytokines Between MIS-C and COVID-19 Pediatric Patients

**Figure 3:**
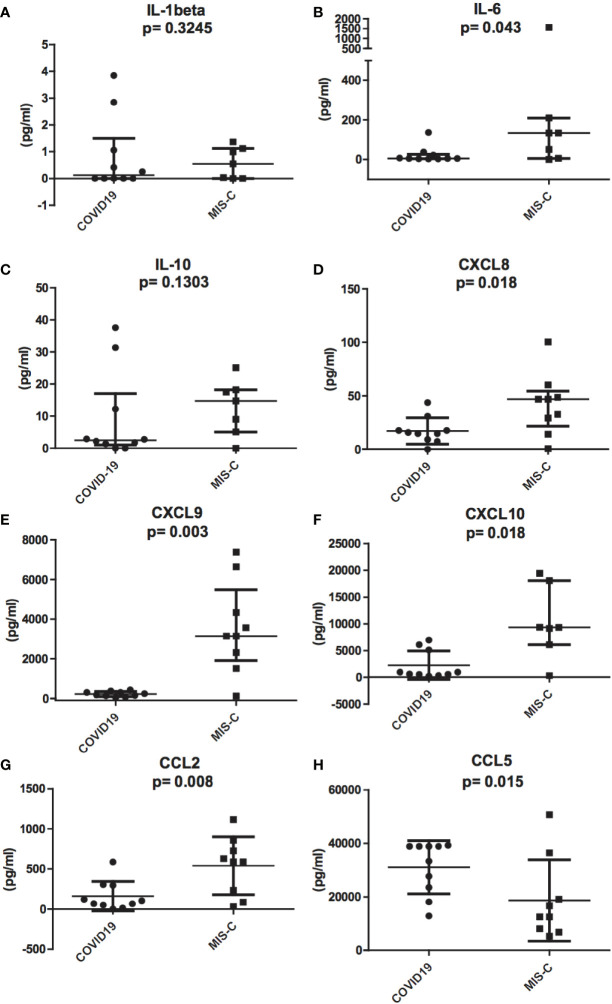
Differences in inflammatory chemokines and cytokines between pediatric patients with COVID-19 (round dots, C1–C10) and MIS-C (squares, M1–M7). Increased levels of IL-1ß, IL-6, IL-8, IL-10, CCL2, CXCL9, and CXCL10 in MIS-C compared to COVID-19 children. **(A)**, IL-1ß, **(B)**, IL-6, **(C)**, IL-10, **(D)**, CXCL8, **(E)**, CXCL9, **(F)**, CXCL10, and **(G)**, CCL2. **(H)**, CCL5 plasma levels are higher in COVID-19 compared to MIS-C. In the scatter plots the internal horizontal lines indicate the mean value and the two horizontal lines across the mean indicate the standard deviation. Statistical analysis was performed by using the Mann-Whitney U test.

To investigate the role of cytokines and chemokines in the inflammatory status of COVID-19 and MIS-C, we measured plasma levels of IL-1ß, IL-2, IL-4, IL-5, IL-6, IL-10, TNF-α, IL-17A, IFN-α, IFN-γ, CXCL10/IP-10, CXCL8/IL-8, CXCL9/MIG, CCL5/RANTES, and CCL2/MCP1. The results of the analysis of these cytokines and chemokines were fully described in **Table 1**. In particular, we found that IL-6 levels were significantly higher in MIS-C (133.8 pg/ml) than COVID-19 (5.22 pg/ml) (p = 0.043; **Figure 3B**). Accordingly, higher levels of the chemokines CXCL8 (46.9 pg/ml [0.72 to 100.34] *vs* 15.3 pg/ml [0 to 39.8], p = 0.018) and CCL2 (590.4 pg/ml [33.8 to 1,116.8] *vs* 105.6 pg/ml [3.23 to 588.5]) were detected in MIS-C than in COVID-19 (p = 0.0086) (**Figures 3D, G**). While IL-1ß and IL-10 levels were not statistically different between the two groups (**Figures 2A, C**).

Even though IFN-γ was undetectable in the plasma of both groups (see above), IFN-γ-induced chemokines CXCL9 and CXCL10 were higher in MIS-C than in COVID-19. CXCL9 was 3,145.5 pg/ml [120.7 to 7381.7] *vs* 210.3 pg/ml [45.7 to 428.5], respectively, p = 0.0014; **Figure 3E**, and CXCL10 was 9,339.1 pg/ml [311.3 to 19,402.4] in MIS-C *vs* 812.1 pg/ml [244.2 to 6,973.4] in COVID-19, p = 0.009; **Figure 3F**.

In contrast, CCL5 levels were significantly higher in the COVID-19 group compared to MIS-C (36,135.7 pg/ml [12,866.5 to 39,280.5] *vs* 12,500.3 pg/ml [5,323.02 to 50,750], p = 0.015; **Figure 3H**).

### Decrease of IL-6, CCL2, CXCL9, and CXCL10 During Therapy

**Figure 4:**
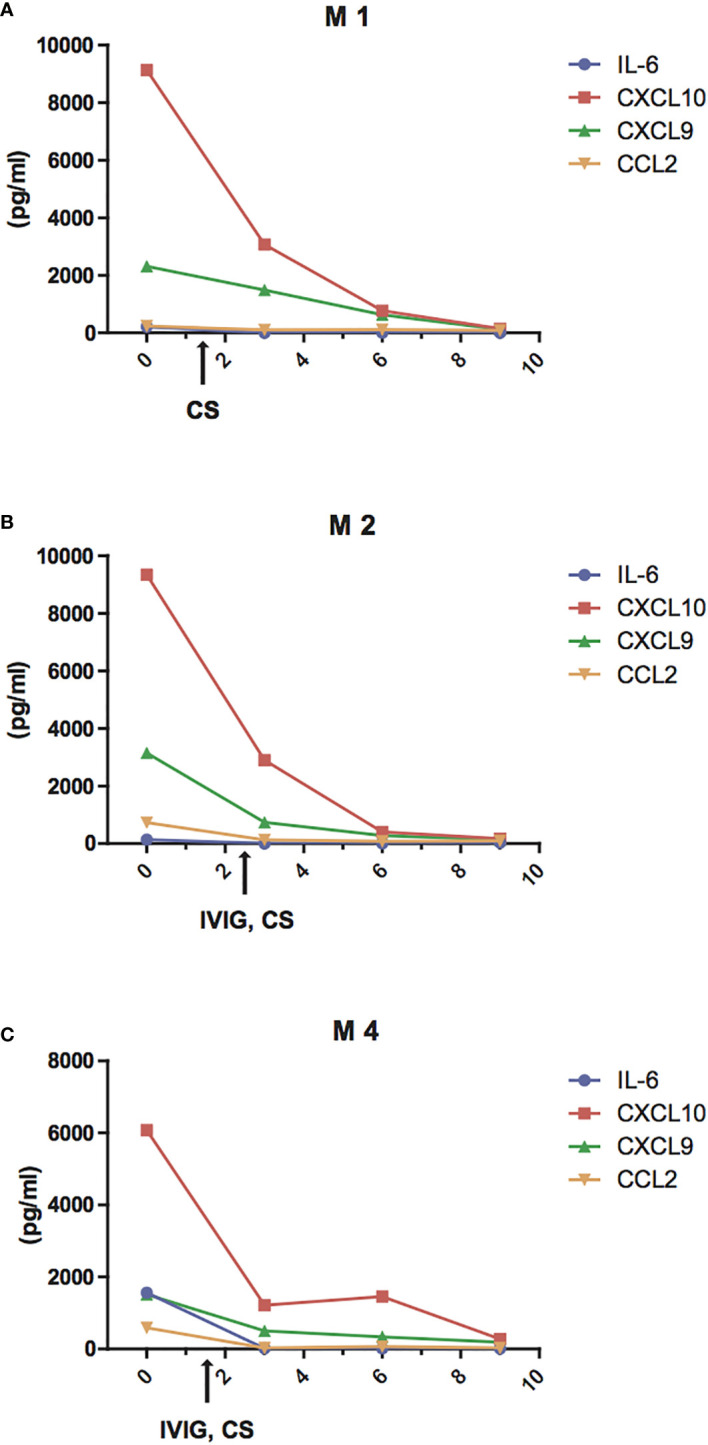
Reduction of IL-6 and chemokine levels during treatment of MIS-C patients. Changes of plasma levels of IL-6 (blue line), CCL2 (orange line), CXCL9 (green line), and CXCL10 (red line) during hospitalization in patients M1 **(A)**, M2 **(B)**, M4 **(C)** admitted for MIS-C, who received high dose IVIG (1 gr/kg in M4 or 2 gr/kg M2) and systemic corticosteroids (methylprednisolone 1 mg/kg i.v. in M4 or 2 mg/kg i.v. both in M1 and M2.)

Next, we evaluated the changes of IL-6 and of the chemokines CCL2, CXCL10, and CXCL9 in three patients with MIS-C who received high dose IVIG (1–2 gr/kg) and systemic corticosteroids (methylprednisolone 1–2 mg/kg i.v.) and successfully recovered in 9 to 13 days. We observed that IL-6, CCL2, CXCL10, and CXCL9 decreased rapidly after the first 2 days of therapy and reached normal levels by 4 to 8 days (**Figures 4A–C**). In particular, the decrease in IL-6 appears faster, returning to normal levels within 3–4 days, while the decrease in CCL2, CXCL10, and CXCL9 occurred after the fifth day of treatment.

## Discussion

Since the beginning of the COVID-19 pandemic, many studies showed that the outcome of SARS-CoV-2 infection in children was much better than in adults (3). Nevertheless, an increasing number of reports from Italy, Europe, and North America questioned these initial observations, describing children with COVID-19-associated multisystem inflammatory conditions (14–16, 18, 27), which is now defined as MIS-C (15). Our study describes the clinical, laboratory, and immunological features of children exposed to SARS-CoV-2 infection, and who had a mild course, compared to children developing MIS-C. The degree of inflammation, as measured by CRP, was significantly greater in MIS-C compared with pediatric COVID-19, as described in other studies (28, 29). Moreover, some of the laboratory findings already described in MIS-C revealed biochemical and immunological features that are also observed in sHLH (14, 30), such as lymphopenia, thrombocytopenia, hypertriglyceridemia, and hyperferritinemia. In children with MIS-C there was a significant reduction of lymphocytes and platelets as compared to the COVID-19 group. Notably lymphopenia and thrombocytopenia are also typical of the hyperinflammatory form of COVID-19 in adults (7, 12).

The analysis of the plasmatic levels of inflammatory chemokines and cytokines in patients with COVID-19 and MIS-C showed higher levels of IL-6, CXCL8, CCL2/MCP-1, CXCL9/MIG, and CXCL10/IP-10 in MIS-C, whereas CCL5 levels were significantly higher in the COVID-19 group. This is probably due to the higher production of CCL5 by epithelial cells during inflammatory airway disorders (31), or to different residual platelets after centrifugation of patients’ plasma, because platelets also represent a significant source of CCL5 (32, 33).

To investigate the association between the clinical features of MIS-C and the rise of these specific inflammatory mediators, we analyzed circulating IL-6, CCL2, CXCL10, and CXCL9 at different time points during the treatment with intravenous methylprednisolone 1–2 mg/kg and high dose IVIG (1–2 gr/kg). In these three patients, we detected a rapid decrease of IL-6, which reversed to normal levels in 3–4 days, while CCL2, CXCL10, and CXCL9 returned to normal levels by the fifth day of the disease. This might account for the results of recent studies showing resistance to immunoglobulin treatment and/or subsequent development of coronary artery aneurysms even after IVIG in MIS-C (28, 34).

The detection of high levels of pro-inflammatory cytokines in MIS-C is similar to what observed in adults with severe COVID-19 (35), where the appearance of respiratory distress coincides with a decline in the viral load in the respiratory tract and the increase in markers of hyperinflammation (36). Although MIS-C and adult “hyperCOVID” are different from a clinical point of view, the common hyperinflammatory signature and the favorable response to immunomodulatory therapies strongly suggest a correlation of MIS-C with an hyperinflammatory response triggered by SARS-CoV-2 infection.

The direct comparison of the immune response in MIS-C and COVID-19 pediatric patients has revealed distinct patterns: while children with typical COVID-19 syndrome displayed a significant increase of IFN-α plasma levels and increased expression of type I IFNs inducible genes ISG15 and IFIT1 in granulocytes and PBMC, patients with MIS-C exhibited low-level expression of ISG15 and IFIT1 mRNAs, as observed in healthy controls. This is consistent with the observation of low levels of pDCs in the peripheral blood of MIS-C patients, as compared to COVID-19 children. Although many cell types can produce IFN-α after viral infections, pDCs are the major source of this cytokine (25, 26). pDCs express receptors for single-stranded RNA or DNA, such as the Toll-like receptor (TLR)-7 and -9 (37, 38), which are essential to sense the viral pathogen and to trigger the innate immune response. Thus, a pivotal role of pDCs in controlling SARS-CoV-2 infection has been hypothesized. In 2007, Luisa Cervantes-Barragan et al. identified pDCs as the major source of type I IFN in response to human SARS-CoV, suggesting an important biologic role of pDC-derived type I IFNs for highly pathogenic coronavirus infections in humans (39). Hadjadj J et al. also observed in adults affected by severe COVID-19 a reduction of pDCs compared to healthy controls (40), probably because pDC activation results in the massive pDC apoptosis caused by IFN-α (41). Likewise, patients with genetic variants of genes related to IFN-α response or carrying anti-IFN-α antibodies display a severe outcome of COVID-19, suggesting a primary role of IFN-α in controlling SARS-CoV-2 infection (42, 43). Moreover, the link between circulating IFN-α and pDCs levels is now well-proven. For instance, patients with IFNGR1 mutations show reduced levels of tissue and circulating pDCs in the acute phase of mycobacterial infection (44). Similarly, low levels of IFN-α are associated with low pDC levels in MIS-C subjects.

There is evidence that coronaviruses can interfere with type I and type III interferon responses, possibly resulting in a hyperinflammatory process in patients who cannot control viral replication or are subjected to high SARS-CoV-2 viral load infection (45, 46). Analysis of COVID-19 patients at different time points during infection revealed a distinct pattern of IFN-α production with sustained/high response in mild-to-moderate COVID-19 patients and low or no response in critically ill patients (40). Moreover, Rowley et al. have suggested that the timing of the type I IFN response to SARS-CoV-2 infection could be related to the extent of viral load and genetic differences in the host response: when viral load is low, IFN-α response appears at an early stage and contributes to viral clearance, resulting in mild manifestations; conversely, when viral load is high and/or genetic factors result in higher susceptibility to SARS-CoV-2, virus replication can affect the extent of IFN-α response resulting in hyperinflammation with lymphopenia and neutrophilia (47).

Analysis of IFN-γ levels has shown that this cytokine is undetectable in the plasma of both MIS-C and COVID-19 children. Nevertheless, patients with MIS-C show higher levels of IFN-γ-related chemokines, CXCL9/MIG and CXCL10/IP-10, suggesting higher production of IFN-γ in children developing MIS-C. Higher levels of CXCL9 and CXCL10 have been observed in patients with sHLH, which are conditions characterized by elevated IFN-γ response and hyperinflammation (48). This suggests that the elevated CXCL9 and CXL10 in MIS-C are probably markers of increased IFN-γ production in lymphoid organs and of secondary induction of pro-inflammatory cytokines and chemokines (e.g., IL-6, CXCL8, CCL2) by macrophages. In addition, the increase of these chemokines in patients with MIS-C might be partially related to the interaction of PAMPs and viral molecules with innate immune receptors, such as TLR3 (49, 50).

Along with lymphopenia and thrombocytopenia, neutrophilia has also been observed in children with MIS-C. Given previous findings on the role of neutrophils on the endothelial and cardiac damage in KD (51, 52) and because the number of polymorphonuclear neutrophils (PMNs) is one factor used in the Kobayashi score in predicting IVIG-non responders, we evaluated neutrophil-derived inflammatory mediators, MPO, ROS, and damaged-DNA levels, as markers of plasma levels of NETs which are involved in the cardiovascular damage (53, 54). The generation of NETs by neutrophils, called NETosis, can be stimulated by many viruses and virus-induced NETs can in turn trigger inflammatory and immunological reactions in an uncontrolled manner, leading to an exaggerated systemic inflammatory response, similar to hyperinflammation seen in both MIS-C and severe COVID-19 (28, 54). As mentioned above, as index of NETs activity, we measured MPO and damaged-DNA levels: MPO presents higher levels in patients with MIS-C and a significant correlation between the levels of MPO and neutrophils has been reported. These findings are consistent with the data shown by Zuo et al. on the neutrophil role in the systemic vascular injury observed in adults with COVID-19 (55). The diffuse microvascular damage seems to have a role in the most severe forms of MIS-C, such as forms of atypical uremic-hemolytic syndrome and the occurrence of shock that, besides cardiac injury, seems to be determined by a capillary leak syndrome (56). Again, neutrophils activation has been demonstrated to be crucial in a form of capillary leak syndrome, suggesting a central role of neutrophils in the pathogenesis of MIS-C (57). All these pieces of evidence suggest a central role of neutrophils in the pathogenesis of MIS-C, possibly through a generalized vascular injury caused by NETosis.

Collectively, our study defines a pattern of distinctive immune response in children with MIS-C. This is characterized by high plasmatic levels of IL-6, of the chemokines CCL2 and CXCL8, which are chemoattractants for monocytes and neutrophils (58, 59) and CXCL9 and CXCL10, suggesting the prevalence of a Th1 type immune response. Increased levels of IFN-γ can actively enhance the inflammatory response of neutrophils as suggested by elevation of MPO levels. In addition, the increase of damaged-DNA and MPO in some patients with MIS-C suggests a possible role of PMNs in inducing microvascular damage due to the exaggerated systemic inflammatory response to SARS-CoV-2. Finally, the profound depletion of pDCs in this group of patients, together with the peculiar immunologic profile, might be useful to identify children with SARS-CoV2 infection who are developing MIS-C.

## Data Availability Statement

The raw data supporting the conclusions of this article will be made available by the authors, without undue reservation.

## Ethics Statement

The studies involving human participants were reviewed and approved by the Ethics Committee of Brescia, protocol NP4000. Written informed consent to participate in this study was provided by the participants’ legal guardian/next of kin. Written informed consent was obtained from the individual(s), and minor(s)’ legal guardian/next of kin, for the publication of any potentially identifiable images or data included in this article.

## Author Contributions

FC has collected clinical data, performed cytokine studies, and written the manuscript. MG has performed analysis of cytokines and contributed to write the manuscript. EG has analyzed plasma for biomechical markers and revised the manuscript. NT has performed transcriptional studies and revised the manuscript. AM has performed analysis of cytokines and revised the manuscript. PP has evaluated and diagnosed the COVID-19 patients and revised the manuscript. ST has evaluated and diagnosed the COVID-19 patients and revised the manuscript. GB has designed the study and revised the manuscript. FZ has evaluated and diagnosed the MISC patients and revised the manuscript. MCa has evaluated and diagnosed the COVID-19 patients and revised the manuscript. DM has performed the lymphocyte subsets study and revised the manuscript. MCh performed the lymphocyte subsets study and revised the manuscript. EC has analyzed ROS and revised the manuscript. GM has contributed to study design and revised the manuscript. MAC has contributed to study design and revised the manuscript. ATa has evaluated and diagnosed the MISC patients and revised the manuscript. ATo has designed the study and revised the manuscript. RB has designed the study and contributed to write the manuscript. All authors contributed to the article and approved the submitted version.

## Funding

This work was supported by grants from Fondazione Cariplo in collaboration with Regione Lombardia and Fondazione Umberto Veronesi (Rif. 2020-1355 and 2020-1376). This work was also supplied by Fondazione Spedali Civili di Brescia.

## Conflict of Interest

The authors declare that the research was conducted in the absence of any commercial or financial relationships that could be construed as a potential conflict of interest.

## Supplementary Material

Supplementary Table 1 Clinical and laboratory features of COVID-19 patients at the time of hospital admission.

Supplementary Table 2 Clinical and laboratory features of MIS-C patients at the time of hospital admission.
