# Supplementary material for: Plasmacytoid Dendritic Cells Depletion and Elevation of IFN-γ Dependent Chemokines CXCL9 and CXCL10 in Children With Multisystem Inflammatory Syndrome
Source: Front Immunol. 2021 Mar 26;12:654587. doi: 10.3389/fimmu.2021.654587 (PMC8033149; doi:10.3389/fimmu.2021.654587)
Supplement: Supplementary Table 1 — Clinical and laboratory features of COVID-19 patients at the time of hospital admission. [file Table_1.docx]

**Supplemental Table 1. Clinical and laboratory features of COVID19 patients at the time of hospital admission**

| Patient | C1 | C2 | C3 | C4 | C5 | C6 | C7 | C8 | C9 | C10 |
| --- | --- | --- | --- | --- | --- | --- | --- | --- | --- | --- |
| Age (years) | <1 | 12 | 8 | 12 | <1 | <1 | <1 | 14 | <1 | 5 |
| Days of disease | 4 | 8 | 2 | 3 | 3 | 2 | 2 | 3 | 3 | 6 |
| Clinical symptoms | Fever, rhinitis, diarrhea | Fever, abdominal pain, diarrhea, rhinitis, cough | Fever | Fever, respiratory distress | Fever, rhinitis, respiratory distress | Fever | Fever, rhinitis | Fever | Fever, cough | Fever, vomiting |
| Hypotension | No | No | No | No | No | No | No | No | No | No |
| Ejection fraction < 55% | - | - | - | - | - | No | No | No | No | - |
| Pericardial effusion | - | - | - | - | - | No | No | No | No | - |
| Interstitial pneumonia | Yes | No | Yes | Yes | Yes | Yes | No | No | Yes | Yes |
| Lymphocytes | 5150 | 1220 | 1636 | 530 | 5560 | 9980 | 7400 | 2400 | 950 | 1300 |
| Neutrophils | 350 | 4430 | - | 1920 | 1320 | 8890 | 1330 | 4640 | 2070 | 4440 |
| Platelets | 353000 | 293000 | 217000 | 183000 | 287000 | 261000 | 475000 | 393000 | 324000 | 257000 |
| CRP mg/L | 7.2 | 47 | 0 | 43.4 | 1.9 | 110.9 | 4.5 | 20.5 | 0 | 86.7 |
| D-dimer ng/mL | - | - | - | 835 | - | 377 | 483 | 201 | - | <200 |
| NT-proBNP ng/L | 610 | 56 | 51 | 116 | 810 | 67 | 110 | 152 | - | - |
| Fibrinogen mg/dL | - | - | - | 423 | 173 | 492 | 296 | 476 | 722 | 425 |
| LDH U/L | 295 | 551 | 167 | 398 | 297 | 290 | 274 | 870 | 287 | - |
| Ferritin mcg/mL | 382 | 456 | 108 | 260 | 123 | 368 | 504 | 290 | 589 | 136 |
| Triglycerides mg/dL | 237 | 131 | - | 64 | 171 | 200 | 115 | 40 | - | - |
| Troponin ng/L | 16 | 5 | 6 | 3 | 16 | 11 | 23 | 5 | 46 | 3 |
| Nasal Swab for SARS-CoV-2 | Positive | Positive | Positive | Positive | Positive | Positive | Positive | Positive | Positive | Positive |
| Nasal Swab for SARS-CoV-2 in parents | Positive | Positive | Positive | Positive | - | - | - | - | Positive | - |
| Exposure to a suspected/ confirmed COVID-19 case within the 4 previous weeks | Yes | Yes | Yes | Yes | Yes | No | - | - | Yes | Yes |
