# Supplementary material for: Plasmacytoid Dendritic Cells Depletion and Elevation of IFN-γ Dependent Chemokines CXCL9 and CXCL10 in Children With Multisystem Inflammatory Syndrome
Source: Front Immunol. 2021 Mar 26;12:654587. doi: 10.3389/fimmu.2021.654587 (PMC8033149; doi:10.3389/fimmu.2021.654587)
Supplement: Supplementary Table 2 — Clinical and laboratory features of MIS-C patients at the time of hospital admission. [file Table_2.docx]

**Supplemental Table 2. Clinical and laboratory features of MIS-C patients at the time of hospital admission**

| Patient | M1 | M2 | M3 | M4 | M5 | M6 | M7 | M8 | M9 |
| --- | --- | --- | --- | --- | --- | --- | --- | --- | --- |
| Age (years) | 7 | 3 | 10 | 14 | 14 | 11 | 4 | 4 | 10 |
| Days of disease | 5 | 7 | 3 | 3 | 1 | 3 | 3 | 1 | 5 |
| Clinical symptoms | Fever, oral mucositis, rash, pharyngitis, lymphadenopathy | Fever, rash, diarrhea, pharyngitis, mild respiratory distress, lymphadenopathy, conjuntivitis | Fever, vomiting, rash, pharyngitis, peeling, conjuntivitis | Fever, oliguria, rigor nucalis | Fever, Chest pain | Fever, abdominal pain, diarrhea, vomiting, rash, pharyngitis, lymphadenopathy, conjuntivitis | Fever, rash, rash, pharyngitis, lymphadenopathy, conjuntivitis | Fever, vomiting, rash, conjuntivitis cheilitis | Fever, abdominal pain, cervical adenopathy, rash |
| Hypotension | Yes | Yes | Yes | Yes | No | Yes | No | Yes | Yes |
| Ejection fraction  < 55% | No | Yes | No | Yes | No | No | No | No | Yes |
| Pericardial effusion | No | No | No | No | Yes | No | No | No | No |
| Interstitial pneumonia | Yes | Yes | Yes | Yes | No | No | Yes | Yes | Yes |
| Lymphocytes | 560 | 940 | 360 | 1370 | 2591 | 620 | 1680 | 250 | 200 |
| Neutrophils | 13950 | 8390 | 6850 | 8000 | 3870 | 3910 | 830 | 4680 | 4077 |
| Platelets | 196000 | 113000 | 143000 | 235000 | 293000 | 166000 | 234000 | 40000 | 112000 |
| CRP mg/L | 264 | 215 | 75 | 338 | 75 | 21 | 6.9 | 148.3 | 227 |
| D-dimer ng/mL | 726 | 685 | 1107 | 1331 | <200 | 2367 | 457 | 4000 | 3100 |
| NT-proBNP ng/L | 13698 | 23981 | - | 27918 | 31 | 1189 | 41 | - | 5970 |
| Troponin ng/L | 80 | 20 | 4 | 328 | 1997 | 3 | 3 | - | - |
| Fibrinogen mg/dL | 737 | 693 | 481 | 1113 | 271 | 425 | 222 | 363 | 575 |
| LDH U/L | 371 | 205 | 282 | 179 | 193 | 239 | 367 | 308 | 237 |
| Ferritin mcg/mL | 644 | 250 | 647 | 525 | 120 | 536 | 80 | 208 | 564 |
| Triglycerides mg/dL | 280 | 209 |  | 76 | 204 | 111 | 95 | - | - |
| Kobayashi score | 6 | 6 | 5 | 8 | 1 | 1 | 4 | 6 | 3 |
| Nasal Swab for SARS-CoV-2 | Weakly positive | Negative | Negative | Negative | Weakly positive | Negative | Negative | Positive | Negative |
| Nasal Swab for SARS-CoV-2 in parents | Negative | Negative | Negative | Negative | Negative | Negative | Positive | Positive | Negative |
| Exposure to a suspected/confirmed COVID-19 case within the 4 previous weeks | Yes | Yes | Yes | Yes | Yes | Yes | Yes | Yes | Yes |
